# Effects of a fruit-vegetable dietary pattern on oxidative stress and genetic damage in coke oven workers: a cross-sectional study

**DOI:** 10.1186/s12940-015-0028-5

**Published:** 2015-05-06

**Authors:** Zheng Xie, Haijiang Lin, Renfei Fang, Weiwei Shen, Shuguang Li, Bo Chen

**Affiliations:** School of Public Health, Peking University Health Science Center, 38 Xueyuan Road, Beijing, 100191 P. R. China; Taizhou City Center for Disease Control and Prevention, 608 The east China sea avenue, Taizhou city, Zhejiang Province 318000 P. R. China; Key Laboratory of Public Health Safety of Ministry of Education, School of Public Health, Fudan University, 138 Yixueyuan Road, Shanghai, 20032 P. R. China

**Keywords:** Coke oven workers, 1-hydroxypyrene (1-OHP), Oxidative stress, Genetic damage, Fruit-vegetable dietary pattern

## Abstract

**Background:**

Coke oven workers (COWs) are exposed to high level of genotoxic chemicals that induce oxidative stress and genetic damage. The dietary intake of certain types of foods may reverse these effects.

**Methods:**

We conducted a cross-sectional study with 51 topside COWs, 79 other COWs, and 67 controls, to assess the effects of dietary patterns on oxidative stress and genetic damage.

**Results:**

Compared to the controls, both topside and other COWs had significantly higher urinary 1-hydroxypyrene levels, serum oxidant levels [malondialdehyde, (MDA)], and genetic damage [micronucleus (MN) frequency & 8-oxo-2′-deoxyguanosine (8-OH-dG)], but lower antioxidant levels [superoxide dismutase (SOD) and glutathione peroxidase, (GPx)]. The fruit-vegetable (FV) dietary pattern was positively correlated with serum SOD levels and negative correlated with serum MDA, MN frequency, and urinary 8-OH-dG. COWs with an FV patter in the highest quartile (Q4) had significantly increased antioxidant levels (SOD and GPx) and decreased oxidant levels (MDA) and genetic damage (MN frequency and 8-OH-dG) than those with an FV pattern in the lowest quartile (Q1).

**Conclusion:**

Compared to control subjects, COWs had increased oxidative stress and genetic damage. A FV dietary pattern may reverse oxidative stress and genetic damage in COWs.

## Background

Coke oven workers (COWs) are exposed to a wide variety of toxic chemicals, including polycyclic aromatic hydrocarbons (PAHs) and benzene series (benzene, toluene and xylenes) [1], which are potentially carcinogenic [2,3]. Epidemiologic studies have shown that COWs have increased incidence of pulmonary cancer due to exposure to these genotoxic chemicals [4].

The compound 8-oxo-2′-deoxyguanosine (8-OH-dG) is a product of DNA oxidation [5]; high exposure to PAHs is associated with high urinary levels of 8-OH-dG [6-8]. At the chromosomal level, the micronucleus (MN) assay detects both clastogenic and aneugenic potentials of genotoxic agents [9]. The MN assay in peripheral blood lymphocytes has been extensively used in Chinese COWs to evaluate chromosomal damage [7,10,11].

Oxidative stress is one of the key factors that contributes to genetic damage [12]. Oxidative stress, which results from an imbalance between reactive oxygen species (ROS) formation and antioxidant activity, leads to damaged lipids, proteins, and cellular macromolecules [13]. In addition to 8-OH-dG [5], malondialdehyde (MDA), superoxide dismutase (SOD), and glutathione peroxidase (GPx) are generally measured to assess oxidation-reduction imbalances [14]. Chinese COWs have been reported to have higher levels of oxidants and lower levels of antioxidants than controls [15].

There are two main methods to reduce oxidative stress and genetic damage in COWs: reduce toxin exposure and inhibit the toxic action of genotoxic chemicals. Fruit and vegetable (FV) consumption has been shown to prevent certain types of cancer [16]. Phytochemicals in FVs may inhibit the genotoxic action of food-borne carcinogens [17]. It has been reported than an adequate intake of fruits, vegetables, and vitamin C is protective in pregnant women exposed to PAHs [18].

Even though carcinogen exposure in COWs is mainly air-borne as opposed to food-borne, we hypothesize that the consumption of FVs in COWs inhibits the genotoxic action of air-borne carcinogens. We performed a cross-sectional study with COWs from a large coking plant in Southern China. Factor analyses were performed to explore the associations between different dietary patterns and parameters of oxidative stress and genetic damage.

## Methods

### Study design and subjects

This study was conducted in September 2009 in a large coking plant in Southern China. The coke oven battery is hollow in the middle; coke oven emissions (COEs) are released when the lid or side door of the coke oven is opened or when the oven battery is not properly sealed. Exposure to COEs depends on several factors including the battery location, i.e., top, side, or bottom of the oven. Topside oven workers are usually exposed to the highest COE levels. In this study, the exposed group consisted of topside workers and other COWs, and the control group consisted of office workers, cafeteria servers, or other members of the coking plant. All COWs were males; therefore, we only selected male subjects for the control group. The working area of the control group was approximately 450 meters away from the coking plant. The control group had a less rigorous working schedule compared to that of COWs.

In this study, we invited 262 workers from the coking plant to participate. Among them, 21 COWs and 16 controls refused to participate, 14 COWs and 10 controls did not provide any blood samples, and 2 COWs and 2 controls did not provide any urine samples. Therefore, 197 subjects were enrolled, including 51 topside COWs, 79 other COWs, and 67 controls. No information was obtained from the 37 subjects who refused to participate. There were no significant differences in demographic characteristics between the participating subjects and the 16 COWs and 12 controls who did not provide any blood or urine samples.

Subjects completed a detailed questionnaire on demographic characteristics such as age, BMI (normal, ≤25; overweight, >25), education level (e.g., high school or below, college, or above), cigarette smoking (nonsmoker or smoker), alcohol consumption (nondrinker or drinker), working hours per day, and years of service. All subjects provided urine and blood samples.

The study was performed with approval from local authorities and the Ethics Committee of School of Public Health at Fudan University, China. Informed consent was obtained from each subject.

### Food frequency questionnaire (FFQ)

Dietary intake information was obtained from a FFQ based on the 2002 China National Nutrition and Health Survey (CNNHS) [19], with slight modifications. The subjects were instructed to report the consumption frequency and amount of 25 food items (as opposed to 33 food items in the CNNHS’s FFQ) within the past three months (as opposed to one year in the CNNHS’s FFQ). Consumption frequency was categorized as daily, weekly, and monthly. Daily consumption of each food item was derived by dividing the weekly consumption by 7 and the monthly consumption by 30. The FFQ was compared with two 24-h recalls (one day 24-h recall at the beginning of a three-month period and one day 24-h recall at the end of the three-month period) performed with 96 adults from Shanghai (median age: 43 y). The results revealed the following intra-class correlation coefficients for macronutrients: total energy, 0.46; total protein, 0.59; total fat, 0.38; carbohydrate, 0.61; dietary fiber, 0.55; and calcium, 0.78.

### Measurement of urinary 1-hydroxypyrene (1-OHP) levels

All participating subjects provided a urine sample (20 ml). Urinary 1-OHP levels were measured by HPLC [20]. The detection limit of 1-OHP was 0.15 ng/ml. In this study, 1-OHP was detected in all urine samples. Urinary 1-OHP levels were adjusted for urinary creatinine excretion (μmol/mol creatinine).

### Measurement of oxidative stress

Blood samples (3 ml) were collected in test tubes devoid of anticoagulant and centrifuged at 1,500 rpm for 10 min. The resulting serum was used to measure SOD, GPx, and MDA levels by enzyme-linked immunosorbent assay (ELISA) (Liansuo Biological Technology Co., Ltd, Shanghai, China).

### Measurement of 8-OH-dG levels

Urinary 8-OH-dG levels were measured by ELISA (Liansuo Biological Technology Co., Ltd). Urinary 8-OH-dG levels were adjusted for creatinine excretion (μmol 8-OH-dG/mol creatinine).

### MN frequency analysis

The cytokinesis-blocked micronucleus (CB-MN) assay was performed as previously described [21]. For the evaluation of chromosomal damage, one scorer blindly analyzed the MN frequencies in 1,000 bi-lymphocytes from each culture.

### Statistical analyses

The SPSS 12.0 (SPSS Inc., Chicago, IL, USA) software package was used for data processing and statistical analyses. Urinary 1-OHP and 8-OH-dG levels were natural log-transformed. All statistical tests were two-sided (α = 0.05). One way ANOVA and chi-square analyses were used to evaluate differences in demographic characteristics and between exposed and control groups. Linear regression models were used to assess the effects of food frequencies and dietary patterns on parameters of oxidative stress and genetic damage.

Factor models using principle components analyses were used to identify dietary patterns. The factors were orthogonally rotated to derive non-correlated factors. In this study, 13–15 factor solutions were specified, and eigenvalues and factor loadings were assessed. Following these analyses, a four-factor solution was selected. Factor loadings were calculated for each food group across the four factors (dietary patterns). For each dietary pattern, a factor score for each subject was calculated by adding the intakes of all food groups weighted by their factor loadings. Each factor was divided into quartiles; sample characteristics were compared between the highest (Q4) and lowest quartiles (Q1) by analysis of covariance (for means) and chi-square test (for frequencies).

## Results

Table 1 shows the demographic characteristics of all participating subjects. Topside workers were younger than control subjects and other COWs. Topside and other COWs had shorter years of service compared to the controls. No other significant differences in demographic characteristics were obtained between COWs and controls.

**Table 1:** **Demographic characteristics of coke oven workers (COWs) and controls**

**Characteristics**	**Controls**	**Topside COWs**	**Other COWs**
	**(n = 67)**	**(n = 51)**	**(n = 79)**
Age (years)^a^	38.1 (7.5)	35.5 (8.3)^*^	37.8 (8.2)
BMI (kg/m^2^)^b^			
≤25	46 (69)	40 (78)	58 (73)
>25	21 (31)	11 (22)	21 (27)
Level of education^b^			
High school and below	57 (85)	46 (90)	73 (92)
College and above	10 (15)	5 (10)	6 (8)
Cigarette smoking^b^			
Nonsmoker	28 (42)	15 (29)	28 (35)
Smoker	39 (58)	36 (71)	51 (65)
Alcohol drinking^b^			
Nondrinker	25 (37)	18 (35)	25 (32)
Drinker	42 (63)	33 (65)	54 (68)
Working hours per day^a^	8.4 (1.1)	7.6 (0.7)	7.7 (0.8)
Years of service^a^	10.2 (3.4)	6.9 (2.3)^***^	7.6 (1.9)^***^

^*^ *P* < 0.05, ^***^ *P* < 0.001 vs. control group.

^a^Arithmetic mean (standard deviation).

^b^Number (%).

COWs, especially topside workers, had significantly higher urinary 1-OHP levels than the controls (Table 2), indicating that COWs were exposed to high levels of PAHs and other COEs. Compared to the controls, both topside COWs and other COWs had significantly lower levels of antioxidants (SOD and GPx) and increased oxidant levels (MDA) and genetic damage (MN frequency and 8-OH-dG; Table 2).

**Table 2:** **Exposure levels, oxidative stress, and DNA damage in coke oven workers (COWs) and controls**

**Parameters**	**Controls**	**Topside COWs**	**Other COWs**
	**(n = 67)**	**(n = 51)**	**(n = 79)**
Exposure levels			
1-OHP(μmol/mol creatinine)^a^	0.6 (0.2–1.9)	6.5 (2.9–10.3)^***^	2.4 (1.1–5.7)^***^
Oxidative stress			
SOD (U/mL)^a^	1068.5 (35.2)	753.7 (60.9)^***^	821.8 (47.1)^***^
GPx (U/mL)^a^	84.2 (2.3)	47.8 (5.6)^***^	55.1 (3.9)^***^
MDA (nmol/mL)^a^	3.9 (0.8)	6.9 (1.4)^***^	6.7 (1.2)^***^
DNA damage			
MN frequency (‰)^a^	2.2 (1.8)	5.1 (4.7)^***^	4.6 (5.2)^***^
8-OH-dG(μmol/mol creatinine)^b^	1.3 (0.8–1.8)	2.3 (1.0–7.1)^***^	2.4 (0.7–6.9)^***^

^***^ *P* < 0.001 vs. control group.

^a^Arithmetic mean (standard deviation).

^b^Geometric mean (95% confidence interval).

Four main dietary patterns were identified by factor analysis: a rice-noodle pattern (Pattern 1), an FV pattern (Pattern 2), a high protein food pattern (Pattern 3), and a snack-sugar pattern (Pattern 4; Table 3). Individual factor loadings were interpreted similarly to correlation coefficients; positive values contributed the most to the factor score and negative values contributed the least to the factor score. The four major dietary patterns explained 7.7% (Pattern 1), 7.3% (Pattern 2), 6.7% (Pattern 3), and 4.9% (Pattern 4) of the variance in dietary intake. Altogether, the four factors explained 26.6% of the variance in dietary intake.

**Table 3:** **Factor loadings for dietary patterns in all subjects**

**Food groups**	**Pattern 1**	**Pattern 2**	**Pattern 3**	**Pattern 4**
Rice	0.81	/	0.21	/
Noodles	0.73	0.19	/	0.21
Steamed buns	/^a^	/	/	−0.16
Coarse cereals	/	−0.25	/	−0.15
Dark vegetables	0.21	0.78	/	/
Light vegetables	0.24	0.70	/	/
Mushrooms	/	/	/	/
Legume products	/	/	0.31	/
Fruit	/	0.76	−0.21	0.15
Eggs	0.18	/	/	−0.17
Pork	0.20	/	0.61	/
Poultry	0.19	0.20	0.59	0.20
Beef and mutton	/	−0.18	/	/
Processed meat	−0.23	/	0.43	−0.23
Visceral organs	−0.18	/	0.27	/
Fish	0.24	0.21	/	0.18
Shrimps and crabs	/	/	−0.17	/
Dairy products	/	0.19	/	/
Beverages	−0.29	/	−0.25	0.23
Alcohol	−0.18	/	0.22	−0.27
Fried pasta	/	−0.23	−0.28	−0.27
Snacks and cookies	−0.15	/	−0.26	0.84
Sugars and preserves	/	−0.19	/	0.61
Oils	0.31	/	/	0.32
Condiments	0.15	/	/	/

^a^Factor loadings ≤ │0.15│ were excluded for simplicity.

Pattern 1: Rice-noodle pattern; Pattern 2: Fruit-vegetable pattern; Pattern 3: High protein foods; Pattern 4: Snack-sugar pattern.

Multivariable regression models were used to assess the effects of different food frequencies and dietary patterns on oxidative stress and genetic damage (Table 4). The adjusted regression was conducted by incorporating all covariates into the model, which included age, BMI, level of education, cigarette smoking, alcohol consumption, working hours per day, years of service, urinary 1-OHP levels, and exposure group (controls, other COWs, and topside COWs).

**Table 4:** **Food frequency and dietary pattern in association with oxidative stress and DNA damage [shown as the standardized coefficients (β) and significance (** ***P*** **)]**

	**Oxidative stress**						**DNA damage**			
	**SOD**		**GPx**		**MDA**		**MN**		**8-OH-dG**	
	**β**	***P***	**β**	***P***	**Β**	***P***	**β**	***P***	**β**	***P***
Frequency of fruit consumption (daily vs. weekly+monthly)										
Crude	0.117	0.035	0.034	0.241	−0.151	0.027	−0.119	0.129	−0.197	0.040
Adjusted^a^	0.104	0.048	0.031	0.246	−0.143	0.027	−0.138	0.105	−0.178	0.051
Frequency of dark vegetable consumption (daily vs. weekly+monthly)										
Crude	0.098	0.064	0.122	0.026	−0.144	0.031	−0.212	0.033	−0.205	0.039
Adjusted^a^	0.095	0.065	0.109	0.041	−0.151	0.025	−0.223	0.036	−0.195	0.042
Frequency of light vegetable consumption (daily vs. weekly+monthly)										
Crude	0.121	0.045	0.084	0.097	−0.132	0.046	−0.175	0.058	−0.188	0.049
Adjusted^a^	0.112	0.052	0.091	0.092	−0.135	0.045	−0.168	0.066	−0.184	0.051
Pattern 1: Rice-noodle										
Crude	−0.037	0.721	−0.082	0.427	0.054	0.596	0.115	0.260	0.038	0.713
Adjusted^a^	0.037	0.396	−0.006	0.885	0.005	0.951	0.090	0.357	−0.025	0.686
Pattern 2: Fruit-vegetable										
Crude	0.109	0.041	0.082	0.106	−0.121	0.031	−0.257	0.017	−0.211	0.031
Adjusted^a^	0.182	0.013	0.099	0.057	−0.130	0.026	−0.293	0.012	−0.220	0.028
Pattern 3: High protein foods										
Crude	−0.074	0.297	−0.062	0.412	−0.004	0.967	0.196	0.083	0.091	0.218
Adjusted^a^	−0.057	0.233	−0.033	0.327	−0.021	0.789	0.174	0.100	0.079	0.164
Pattern 4: Snack-sugars										
Crude	−0.013	0.900	0.009	0.932	−0.100	0.330	0.033	0.696	0.034	0.739
Adjusted^a^	−0.031	0.465	−0.036	0.339	−0.072	0.395	0.040	0.583	0.072	0.258

The food frequency comparison was within the dichotomized variables. The dietary pattern comparison was between the highest quartile subjects (Q4) and the lowest quartile subjects (Q1).

^a^Adjusted for age, BMI, level of education, cigarette smoking, alcohol consumption, working hours per day, years of service, urinary 1-OHP levels, and exposure group (controls, other COWs, and topside COWs).

In the food frequency analysis, the consumption frequency of each food item was dichotomized before being incorporated into the regression model. Daily fruit consumption was associated with significantly higher antioxidant levels (SOD) and lower oxidant levels (MDA) and genetic damage (8-OH-dG) compared to weekly or monthly fruit consumption, both in crude and adjusted models (Table 4). Similar results were obtained with dark vegetable consumption (positively correlated with high GPx levels, and negatively with MN frequency and 8-OH-dG) and light vegetable consumption (positively correlated with high SOD levels, and negatively with MDA and 8-OH-dG).

In the dietary pattern analysis, only the FV pattern was associated with increased antioxidant levels (SOD) and decreased oxidant levels (MDA) and genetic damage (MN frequency and 8-OH-dG), both in the crude and adjusted models (Table 4). High serum levels of GPx were positive correlated with the FV pattern in the adjusted model; however, the results were not significant (*P* < 0.1). Similarly, high MN frequency was positively correlated with the high protein food pattern in both the crude and adjusted models (*P* < 0.1).

**Figure 1:**
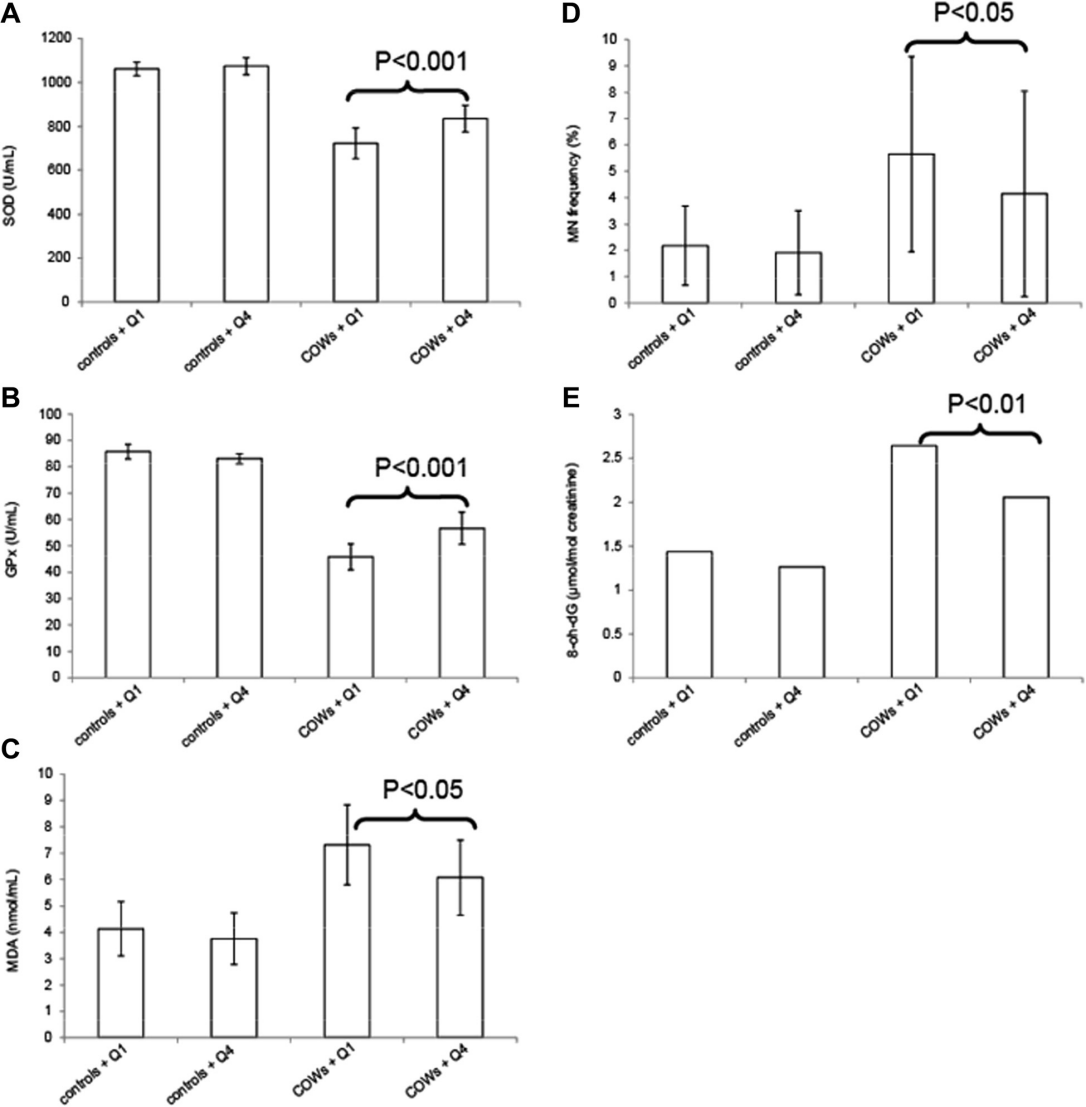
Oxidative stress and genetic damage in association with occupational exposure and FV consumption. SOD **(A)**, GPx **(B)**, and MDA **(C)** levels were measured to assess oxidative stress; MN frequency **(D)** and 8-OH-dG **(E)** levels were measured to assess genetic damage. SOD, GPx, MDA, and MN frequency are expressed as arithmetic mean ± standard deviation, 8-OH-dG levels are expressed as geometric mean. The above parameters were compared among four groups: control subjects with an FV pattern in the lowest quartile (controls+Q1), control subjects with an FV pattern in the highest quartile (controls+Q4), exposed subjects with an FV pattern in Q1 (COWs+Q1), and exposed subjects with an FV pattern in Q4 (COWs+Q4).

To clearly demonstrate the effects of FV intakes, oxidative stress and genetic damage were compared amongst different exposure groups and dietary patterns. Both topside COWs and other COWs were merged together to increase statistical power. The results revealed no significant differences in the control group (Figure 1). However, COWs with an FV in the highest quartile (Q4) had significantly increased antioxidant levels (SOD and GPx) and decreased oxidant levels (MDA) and genetic damage (MN frequency and 8-OH-dG) than those with an FV pattern in the lowest quartile (Q1).

## Discussion

In this study, COWs were exposed to high levels of 1-OHP, a biomarker of PAH exposure. Topside COWs had significantly higher urinary 1-OHP levels than other COWs; therefore, the topside oven is the most polluted area in a coking plant.

COWs had low levels of antioxidants (SOD and GPx) and high levels of oxidants (MDA), which contributed to an imbalance in oxidation-reduction reactions. This imbalance may be caused by a high exposure to pollutants in this study. COWs have been exposed to several PAHs and benzene series, which are converted into ROS [22,23].

Oxidative stress is a common mechanism by which some environmental chemicals induced damage. Environmental pollutants stimulate a variety toxic mechanisms at the molecular level; oxidative stress seems to be the common denominator leading to damage of cellular membranes, DNA, and, proteins [24]. As a biomarker of oxidative DNA damage, urinary 8-OH-dG levels were nearly 2× higher in the exposed workers than in the control subjects. Therefore, the COWs surveyed in this study had a higher burden of oxidative stress, which contributed to DNA damage. The fact that MN frequency, which is widely used in the detection of different types of genetic damage in eukaryotic cells, was significantly higher in COWs than in control subjects, reveals that the exposure level in this study was capable of inducing genetic damage. Studies have reported excessive oxidative stress and genetic damage in COWs with a chemical exposure level similar to or lower than that in our study [6-8,10,11]. The results obtained in this study suggest that it is urgent to control the exposure level to COEs in China.

The most interesting finding in our study was that the consumption of FVs enhanced antioxidant levels (SOD and GPx) and reduced oxidant levels (MDA) and genetic damage (8-OH-dG and MN frequency). In spite of well documented in vitro and in vivo evidence on the antioxidant effects of FVs, most intervention studies have failed to observe positive effects in humans [25,26]. Chang et al. [25] who evaluated the effects of 10 FV servings/day for two weeks on endogenous DNA damage, reported no significant effects relative to the control group. Briviba et al. [26], who used an RCT design to study the effects of two daily FV servings for four weeks on the health outcomes of 64 nonsmoking male subjects, reported no significant effects relative to the control group. It should be noted that both of the above studies had limitations. In Chang’s study, only 28 subjects were investigated, while in the study by Brivida et al., subjects were healthy, well-nourished, nonsmoking men. In this study, the COWs were exposed to higher levels of toxic chemicals than the subjects in Briviba’s study.

Similar to our findings, two recent studies have also reported a protective effect of FVs on oxidative stress and DNA damage [18,27]. In a Korean study involving 715 pregnant women [18], there was a positive correlation between urinary MDA and 1-OHP levels and low FV consumption. In a European study involving 229 mothers and 612 newborns [27], DNA adduct levels in cord blood were negatively correlated with birth weight; the correlation was more pronounced in mothers who had low FV intakes. There are two possible reasons than can explain the results of the above two studies. Firstly, the protective effects of FVs may be attributed to the presence of vitamin C, vitamin E, carotenoids including β-carotene, and phytochemicals [28,29]. Secondly, populations with high FV intakes tend to consume low levels of meat, thereby reducing their dietary exposure to mutagenic agents (e.g., PAHs and heterocyclic amines). The second explanation is not applicable in our study, because the COWs were exposed to much higher levels of mutagenic agents from the coking environment than from their dietary sources. Thereby, our data probably revealed that the antioxidant constituents in FVs had protective effects on oxidative stress and genetic damage.

Our study had some limitations. Firstly, the use of lymphocytes as a surrogate tissue for the measurement of DNA damage is not optimal; it is possible that FVs have more physiological effects in tissues other than in circulating lymphocytes. Secondly, biological samples for measuring oxidative stress and genetic damage were only collected once; therefore, the results may not be reproducible. Thirdly, COWs were exposed to several types of genotoxic chemicals. Therefore, a better exposure biomarker would have been benzo[a]pyrene DNA adducts as opposed to 1-OHP. Fourthly, the FV pattern had *P* values less than 0.05 but higher than 0.01 in certain subjects. It is apparent that the sample size in our study was too small. Future studies with larger sample size should be performed.

## Conclusion

COWs were exposed to high levels of 1-OHP, a biomarker of PAH exposure, and had low antioxidant levels (SOD and GPx) and high oxidant levels (MDA) and genetic damage (MN frequency and 8-OH-dG). The FV dietary pattern had beneficial effects on reversing oxidative stress and genetic damage in COWs.

## Abbreviations

1-OHP: 1-Hydroxypyrene
8-OH-dG: 8-oxo-2′-deoxyguanosine
BMI: Body mass index
CB-MN: Cytokinesis-blocked micronucleus
COEs: Coke oven emissions
COWs: Coke oven workers
DNA: Deoxyribose nucleic acid
ELISA: Enzyme-linked immunosorbent assay
FFQ: Food frequency questionnaire
GPx: Glutathione peroxidase
HPLC: High performance liquid chromatography
MDA: Malondialdehyde
MN: Micronucleus
ROS: Reactive oxygen species
SOD: Superoxide dismutase
